# Using an optimality model to understand medium and long-term responses of vegetation water use to elevated atmospheric CO_2_ concentrations

**DOI:** 10.1093/aobpla/plv060

**Published:** 2015-05-27

**Authors:** Stanislaus J. Schymanski, Michael L. Roderick, Murugesu Sivapalan

**Affiliations:** 1 Department of Environmental Systems Science, Swiss Federal Institute of Technology Zurich, Universitätstrasse 16, 8092 Zurich, Switzerland; 2 Formerly at: Max Planck Institute for Biogeochemistry, Jena, Germany; 3 Research School of Earth Sciences and Research School of Biology, Australian National University, Canberra 2601, Australia; 4 Australian Research Council Centre of Excellence for Climate System Science, Canberra 2601, Australia; 5 Department of Geography and Geographic Information Science, University of Illinois at Urbana-Champaign, Urbana, Illinois, USA; 6 Department of Civil and Environmental Engineering, University of Illinois at Urbana-Champaign, Urbana, Illinois, USA

**Keywords:** Adaptation, ecohydrology, evapotranspiration, global change, optimality, vegetation

## Abstract

Vegetation has different adjustable properties for adaptation to its environment. Examples include stomatal conductance at short time scale (minutes), leaf area index and fine root distributions at longer time scales (days–months) and species composition and dominant growth forms at very long time scales (years–decades–centuries). As a result, the overall response of evapotranspiration to changes in environmental forcing may also change at different time scales. The vegetation optimality model simulates optimal adaptation to environmental conditions, based on the assumption that different vegetation properties are optimized to maximize the long-term net carbon profit, allowing for separation of different scales of adaptation, without the need for parametrization with observed responses. This paper discusses model simulations of vegetation responses to today's elevated atmospheric CO_2_ concentrations (eCO_2_) at different temporal scales and puts them in context with experimental evidence from free-air CO_2_ enrichment (FACE) experiments. Without any model tuning or calibration, the model reproduced general trends deduced from FACE experiments, but, contrary to the widespread expectation that eCO_2_ would generally decrease water use due to its leaf-scale effect on stomatal conductance, our results suggest that eCO_2_ may lead to unchanged or even increased vegetation water use in water-limited climates, accompanied by an increase in perennial vegetation cover.

## Abstract (precis)

Modelling results presented in this paper suggest that elevated atmospheric CO_2_ concentrations (eCO_2_) lead to reductions in leaf-level stomatal conductance and increases in water use efficiency. One consequence was a predicted increase in the perennial vegetation cover and rooting depth in water-limited regions, which provides further support to the view that the globally observed advancement of woody plants is enhanced by fossil fuel emissions. Simulated vegetation responses to eCO_2_ depend on time scale and climate, but generally compare well with free air CO_2_ enrichment experiments (FACE).

## Introduction

Elevated atmospheric CO_2_ concentrations (eCO_2_) are generally expected to lead to reductions in stomatal conductance and hence leaf-scale water use (Wong *et al*. 1979; Drake *et al*. 1997). This physiological response has been incorporated into many land surface models, allowing to account for the ‘physiological effect’ of eCO_2_ on surface temperatures in addition to the ‘radiative effect’ (Sellers *et al*. 1996; Cao *et al*. 2010). Several modelling studies have concluded that the physiological effect of eCO_2_ on stomata may have resulted in regional and global shifts in the water balance and a general increase in river runoff (e.g. Gedney *et al*. 2006; Betts *et al*. 2007; Gopalakrishnan *et al*. 2011). However, other modelling studies reported that the leaf-scale effect may be offset by concurrent changes in leaf area index, dampening the reduction in vegetation water use due to eCO_2_ (Piao *et al*. 2007; Wu *et al*. 2012; Niu *et al*. 2013) and implicated land use change or changes in solar irradiance as possible reasons for increases in continental river runoff (Oliveira *et al*. 2011). So far, there is only limited empirical evidence for the full range of vegetation responses to eCO_2_, but both theoretical considerations and remote sensing data have led some authors to link the observed global increase in perennial vegetation cover (‘woody thickening’) to increasing atmospheric CO_2_ concentrations (*C*_a_) (Bond and Midgley 2000, 2012; Berry and Roderick 2002; Eamus and Palmer 2008; Donohue *et al*. 2013), suggesting that stomatal closure is indeed only the first step in a long cascade of potential effects of eCO_2_. These may include alterations in species compositions, perennial vegetation cover and rooting depths, which come about as the amount of transpiration required to fix a given amount of CO_2_ declines with increasing atmospheric CO_2_ concentrations. Such alterations are likely to only become obvious after several generations of plants, which, for perennial plants, can take decades to centuries or beyond.

Large-scale free-air CO_2_ enrichment (FACE) experiments allow separation of the *C*_a_ effect on different plant species from other environmental changes, which is very difficult for remote sensing observations. However, the first FACE experiments were only launched in the 1990s, focussing mainly on temperate ecosystems (Ainsworth and Long 2005), and most of them have come to an end already (Norby and Zak 2011), as they were not intended for the study of long-term vegetation dynamics in response to eCO_2_. The present study investigates whether eCO_2_ might affect vegetation and the water balance differently in the medium and long term using a previously tested model that incorporates dynamic feedbacks between natural vegetation and the water balance (Schymanski *et al*. 2009*b*). Rather than prescribing vegetation response to environmental change, the model is based on the assumption that vegetation self-optimizes to maximize its ‘Net Carbon Profit’ (i.e. maximizing the difference between carbon acquired by photosynthesis and carbon spent on maintenance of the organs involved in its uptake) and finds the ‘optimal’ vegetation for given environmental conditions. Here we use this model to investigate the different time scales of vegetation response to eCO_2_.

We selected four study sites ranging from dry (water-limited) to wet (energy-limited) conditions in Australia. At each site, we use the model to solve for the optimal vegetation under an assumed climate-CO_2_ combination. We use the model runs to ask the following questions:

1. What would be the difference in predicted annual transpiration rates if only quickly varying vegetation properties (sub-annual scale) were allowed to respond to eCO_2_ (medium-term response)?
2. What would be the difference in predicted annual transpiration rates if all vegetation properties were allowed to respond to increased CO_2_ (long-term response)?
3. Does an increase in atmospheric CO_2_ have similar effects on transpiration in all four catchments and climates for both the medium and long-term responses?

## Methods

### Vegetation optimality model

The model used in this study (vegetation optimality model, VOM) is a coupled water balance and vegetation dynamics model, which does not rely on any input of site-specific vegetation properties or past observations of vegetation response to environmental forcing. This model has been described elsewhere in detail (Schymanski *et al*. 2008*b*, 2009*b*) and the model code is available online (https://github.com/schymans/VOM). In summary, the VOM consists of a physically based multi-layer soil water balance model (0.5 m thick soil layers down to an impermeable bedrock in this study) interfacing with a root water uptake model, which again interfaces with a tissue water balance and leaf gas exchange model. Water fluxes between soil layers and into the fine roots are formulated as functions of water potential gradients and resistances, while leaf gas exchange is simulated as a function of stomatal conductance and leaf-air mole fraction differences. The leaf-internal sink strength for CO_2_ is modelled based on a biochemical model of photosynthesis (von Caemmerer 2000), but simplified by omitting carboxylation-limited conditions (see **Supporting Information** or Schymanski 2007; Schymanski *et al*. 2009*b*). For the present study, the soil water balance model was also simplified in that the catchment was represented by a rectangular block of soil rather than a linear hillslope as in Schymanski (2007) and Schymanski *et al*. (2009*b*). This was found necessary to improve consistency and robustness while parameterizing different catchment geometries (see **Supporting Information** for details).

### Optimality, adjustable vegetation properties and associated trade-offs

The VOM approach is based on the assumption that natural vegetation has co-evolved with its environment over a long period of time leading to a composition that is optimally adapted to the conditions. Optimal adaptation is simulated by allowing dynamic adjustments of different vegetation properties at different time scales:

1. Foliage projective cover and max. rooting depth of perennial plants (decades)
2. Water-use strategies (decades)
3. Foliage projective cover of seasonal plants (daily)
4. Photosynthetic capacity and vertical fine root distributions (daily)
5. Canopy conductance (hourly)

The different vegetation properties are optimized to maximize the community long-term net carbon profit (NCP), i.e. leaf CO_2_ uptake minus respiration costs due to maintenance and turnover of foliage, wood and roots (Schymanski 2007; Schymanski *et al*. 2009*b*).

The canopy is represented by two ‘big leaves’. One big leaf of invariant size (*M*_A,p_, m^2^ big-leaf area m^−2^ ground area) represents perennial vegetation and another big leaf of varying size (*M*_A,s_, m^2^ big-leaf area m^−2^ ground area) represents seasonal vegetation. As the big leaves are not assumed to transmit any light, no overlap between these two leaves is allowed, so that *M*_A,s_ + *M*_A,p_ ≤ 1. The seasonal vegetation is allowed to vary in its spatial extent (*M*_A,s_), but has a limited maximum rooting depth (*y*_r,s_ = 1 m), while the perennial component has optimized but invariant *M*_A,p_ and rooting depth (*y*_r,p_). Maximum rooting depths are assumed to be invariant in time, but the distribution of roots within each root zone is allowed to vary on a day-by-day basis. The photosynthetic capacity in each big leaf (represented by electron transport capacity, *J*_max25_) is also allowed to vary from day to day, while stomatal conductivity (*g*_s_) in each big leaf is allowed to vary on an hourly scale.

The costs and benefits in terms of NCP associated with the optimized parameters in the VOM can be separated into direct and indirect costs and benefits. The direct benefits relate to an increase in photosynthesis, e.g. by increasing big-leaf size, photosynthetic capacity or stomatal conductance. The direct costs relate to increased respiration, e.g. by increased maintenance respiration related to an increased photosynthetic capacity. The indirect benefits relate to carbon gains and losses at a later time, e.g. the consequence of increased stomatal conductance can be a prolonged period of drought-induced stomatal closure and reduced photosynthesis later. Another example is an increase in rooting depth, which has a direct maintenance cost but only an indirect benefit of allowing greater stomatal conductivity and photosynthesis during drought periods. To maximize photosynthetic carbon uptake (*A*_g_) with a limited amount of water, transpiration should be controlled by stomata in such a way that the slope between CO_2_ uptake and transpiration (∂*E*_t_/∂*A*_g_) is kept constant during a day (Cowan and Farquhar 1977; Cowan 1982, 1986; Schymanski *et al*. 2008*a*). This slope is denoted by *λ*_s_ and *λ*_p_ for seasonal and perennial vegetation, respectively. Over longer time periods, the parameters *λ*_s_ and *λ*_p_ should be sensitive to the availability of soil water and this sensitivity could be seen as a plant physiological response shaped by evolution to suit a given environment (Cowan and Farquhar 1977; Cowan 1982). In the VOM, the sensitivity of *λ*_s_ and *λ*_p_ to soil water is parametrized as

(1) $$\lambda_{s}=c_{\lambda f,s}{\left(\sum_{i=1}^{i_{r,s}}h_{i}\right)}^{c_{\lambda e,s}}$$

and

(2) $$\lambda_{p}=c_{\lambda f,p}{\left(\sum_{i=1}^{i_{r,p}}h_{i}\right)}^{c_{\lambda e,p}}$$

where *h* denotes the matric suction head in the soil while *i*_r,s_ and *i*_r,p_ denote the deepest soil layer accessed by roots of seasonal and perennial plants, respectively, while the summation is performed over all soil layers (*i*) within the rooting zone. The parameters *c_λ_*_f,s_, *c_λ_*_e,s_, *c_λ_*_f,p_ and *c_λ_*_e,p_ are assumed to represent the long-term adaptation of a plant community to its environment and are likely influenced by the species composition of the community.

### Separation of medium and long-term responses

Using meteorological data over 30 years, long-term adaptation of vegetation to the environment is modelled by the optimization of six parameters (*M*_A,p_, *y*_r,p_, *c_λ_*_f,p_, *c_λ_*_e,p_, *c_λ_*_f,s_ and *c_λ_*_e,s_) to maximise NCP. The optimization is performed using the shuffled complex evolution (Duan *et al*. 1993, 1994; Muttil and Liong 2004), which searches the parameter space for the global optimum by re-running the 30-year simulation repeatedly with different parameter values. During each run, electron transport capacity of seasonal (*J*_max25,s_) and perennial plants (*J*_max25,p_), vegetated surface area covered by seasonal plants (*M*_A,s_) and the root surface areas of perennial and seasonal plants (*S*_Ar,p_ and *S*_Ar,s_, respectively) are optimized dynamically on a day-by-day basis. For a more detailed description of the optimization algorithms, see Schymanski (2007) and Schymanski *et al*. (2009*b*).

The same 30 years of meteorological forcing for each site were used in combination with different atmospheric CO_2_ concentrations (*C*_a_ = 317, 350 and 380 ppm, representing the observed *C*_a_ values in 1960, 1990 and 2005, respectively). The response to eCO_2_ was then taken as the difference between the results for 350 or 380 and 317 ppm and simulated responses at different sites were compared to answer Question 3 in the Introduction.

Medium-term responses (Question 1 in the Introduction) were simulated by taking the *C*_a_ = 317 ppm simulations and re-running with *C*_a_ = 350 ppm and *C*_a_ = 380 ppm, while only allowing optimization of those vegetation properties that were assumed to vary at seasonal and shorter time scales (root surface areas, stomatal conductances and electron transport capacities). In other words, medium-term response refers to simulations where those variables marked as ‘Constant’ in Table 1 were optimized for *C*_a_ = 317 ppm, while all other variables were optimized for *C*_a_ = 350 ppm or *C*_a_ = 380 ppm.

**Table 1.** Optimized vegetation properties in the VOM and their assumed time scales of variation. Subscripts p and s denote perennial and seasonal vegetation, respectively. Canopy conductance is optimized indirectly, as it depends on environmental conditions, *J*_max25_ and *λ*, the latter of which is determined by the *c_λ_*_…_ parameters using Eqs. (1) and (2).

Symbol	Description	Dynamics
*c_λ_*_e,p_	Exponent of water-use function (perennial veg.)	Constant
*c_λ_*_e,p_	Exponent of water-use function (seasonal veg.)	Constant
*c_λ_*_f,p_	Factor of water-use function for (perennial veg.)	Constant
*c_λ_*_f,s_	Factor of water-use function for (seasonal veg.)	Constant
*G*_s,p_	Canopy conductance to CO_2_ (perennial veg.)	Hourly
*G*_s,s_	Canopy conductance to CO_2_ (seasonal veg.)	Hourly
*J*_max25,p_	Electron transport capacity at 25 °C (perennial veg.)	Daily
*J*_max25,s_	Electron transport capacity at 25 °C (seasonal veg.)	Daily
*M*_A,p_	Fractional cover perennial big leaf	Constant
*M*_A,s_	Fractional cover seasonal big leaf	Daily
*S*_Ar,p_	Fine root surface area per soil volume (perennial veg.)	Daily
*S*_Ar,s_	Fine root surface area per soil volume (seasonal veg.)	Daily
*y*_r,p_	Maximum rooting depth (perennial veg.)	Constant
*λ*_p_	Slope of *E*_t_(*A*_g_)-curve (perennial veg.)	Daily
*λ*_s_	Slope of *E*_t_(*A*_g_)-curve (seasonal veg.)	Daily

To simulate long-term adaptation (Question 2 in Introduction), optimization of all vegetation parameters in Table 1 was performed independently under each *C*_a_ level for each site.

### Study sites and site-specific data

**Figure 1.**
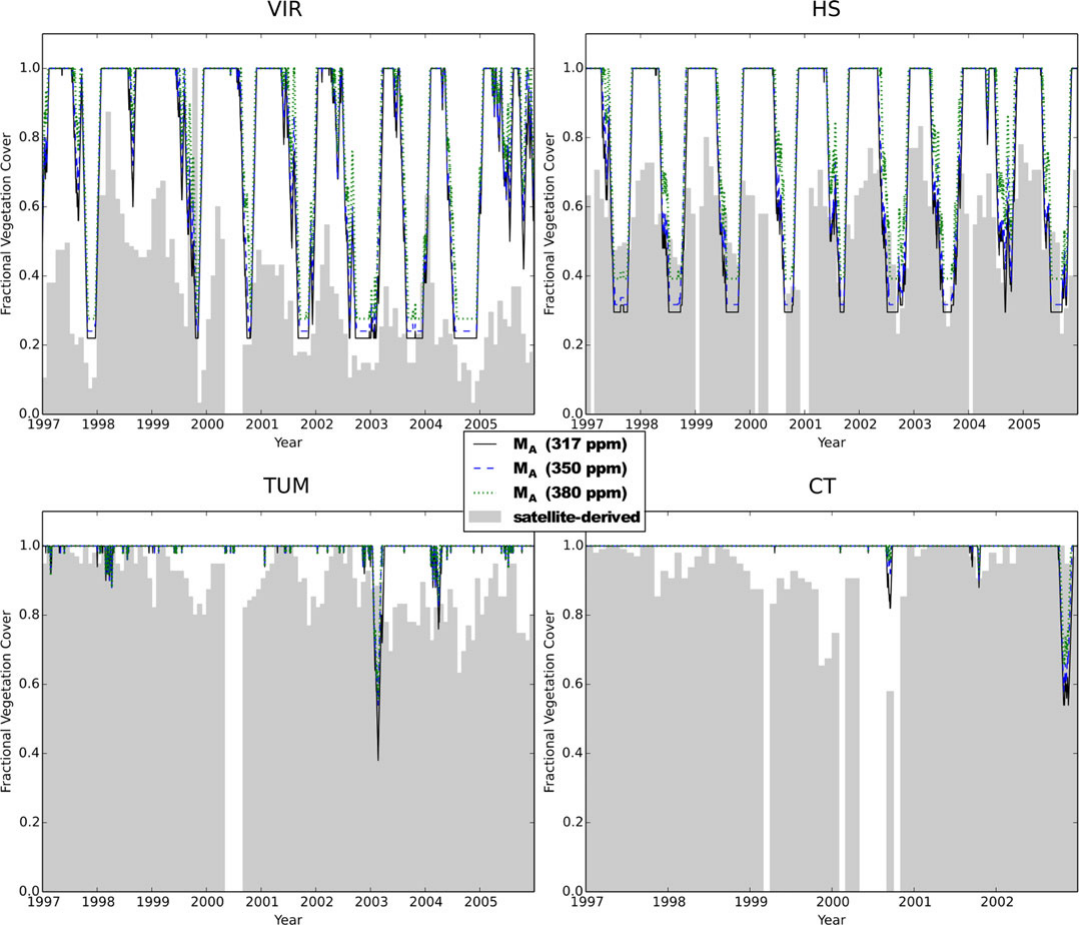
Simulated and satellite-derived FPC at the different sites. Simulation results taken from long-term adaptation runs at 317 (solid lines), 350 (dashed lines) and 380 ppm atmospheric CO_2_ concentrations (dotted lines), satellite-derived (AVHRR) estimates of fractional foliage cover (grey shaded) derived from Donohue *et al*. (2008). Note that gaps in the satellite-derived FPC in year 2000 are due to missing data, not catastrophic events.

The four study sites chosen were all part of the OzFlux network [Ozflux is the Australian and New Zealand Flux Research and Monitoring Network (http://www.dar.csiro.au/lai/ozflux/index.html), which is part of a global network coordinating regional and global analysis of observations from micro-meteorological tower sites (Fluxnet, http://www.fluxnet.ornl.gov/fluxnet/index.cfm)]. The sites span a climatic gradient from semi-arid to humid. The OzFlux sites are long-term monitoring sites for canopy scale CO_2_ and water vapour exchange. These sites were Virginia Park (VIR) and Cape Tribulation (CT) in Queensland, Tumbarumba (TUM) in New South Wales and Howard Springs (HS) in the Northern Territory. The geographic locations, vegetation types and key climatic properties of the different sites are summarized in Table 2, while satellite-derived dynamics of foliage projective cover (FPC, fraction of ground area occupied by vertical projection of foliage) is illustrated in Fig. 1. Catchment and soil properties at the different sites are given in Tables 3 and 4.

**Table 2.** Locations and general conditions of the investigated sites. *E*_p_, net radiation (*I*_n,a_) divided by latent heat of vaporization (*λ*_E_).

Site	Name	Latitude, longitude	Vegetation	Annual rainfall	Annual *E*_p_ (= *I*_n,a_/*λ*_E_)
VIR	Virginia Park	19°53′S, 146°33′E	Open woodland Savanna	580 mm	1810 mm
HS	Howard Springs	12°30S, 131°09′E	Open forest Savanna	1719 mm	1876 mm
TUM	Tumbarumba	35°39′S, 148°09′E	Wet sclerophyll forest	1288 mm	1155 mm
CT	Cape Tribulation	16°06′S, 145°27′E	Tropical rain forest	4097 mm	2085 mm

**Table 3.** Site-specific input data. *Z*, average soil surface position above bedrock; *z*_r_, average channel elevation above bedrock; *γ*_0_, slope angle near drainage channel.

Site	Soil type	Catchment structure (*Z*, *z_r_*, *γ*_0_)
VIR	Sandy loam	15 m, 5 m, 2°
HS	Sandy loam	15 m, 10 m, 2°
TUM	Loam	30 m, 5 m, 11.5°
CT	Sandy clay loam	15 m, 5 m, 2°

**Table 4.** Van Genuchten parameters for the different soil types (Carsel and Parrish 1988). *θ*_r_, residual volumetric water content; *θ*_s_, saturated water content; *α*_vG_, inverse of air entry suction; *n*_vG_, measure of pore size distribution; *K*_sat_, saturated hydraulic conductivity.

Texture	*θ*_r_	*θ*_s_	*α*_vG_ (m^−1^)	*n*_vG_	*K*_sat_ (m s^−1^)
Sandy loam	0.065	0.41	7.5	1.89	1.228 × 10^−5^
Loam	0.078	0.43	3.6	1.56	2.889 × 10^−6^
Sandy clay loam	0.1	0.39	5.9	1.48	3.639 × 10^−6^

Meteorological data for the sites were obtained from the Queensland Department of Natural Resources, Mines and Water [SILO Data Drill (http://www.nrm.qld.gov.au/silo)]. The data set contained, among others, daily totals of global solar radiation, precipitation, and class A pan evaporation, daily maxima and minima of air temperature and daily values for atmospheric vapour pressure, all of which were obtained by interpolation of data from the nearest measurement stations and/or estimated based on proxy data. The methodology used for the compilation of the data set is described in Jeffrey *et al*. (2001). Daily rainfall was distributed evenly over 24 h, while global irradiance (*I*_g_) and air temperature (*T*_a_) were transformed into hourly values by adding diurnal variation as described in **Supporting Information**. The photosynthetically active photon flux density (*I*_a_, mol quanta m^−2^ s^−1^) was obtained from global irradiance (*I*_g_, W m^−2^) using a conversion coefficient of 4.57 × 10^−6^ mol J^−1^ (Thimijan and Heins 1983).

## Results

### Simulated and observed FPC dynamics

Mean FPC (the sum of the area fractions covered by the perennial and seasonal big leaves, *M*_a,s_ + *M*_a,p_) responded positively to eCO_2_ in all simulations (Table 5), but obviously to a very small extent where FPC was already close to 1 at low *C*_a_ (TUM and CT). At these light-limited sites, the model simulated an unexpectedly (and unrealistic) low perennial fractional cover (*M*_A,p_) of 0.28–0.29 for TUM and 0.24–0.25 for CT. On the other hand, simulated seasonal fractional cover (*M*_A,s_) was high and largely invariant at these sites, resulting in almost full cover when both seasonal and perennial fractional covers were combined (Fig. 1). Figure 1 also illustrates that the simulations do capture seasonality and inter-annual variability of satellite-derived FPC estimates at the drier sites (e.g. years 1998–99 at VIR or 2003–04 at HS), and the lack of seasonality at the wetter sites (TUM and CT). However, the model predicts full cover on many occasions when satellite-derived FPC is below 0.8 or even 0.5 (at VIR). Note that the simulations under eCO_2_ suggest a clear increase in perennial FPC (base lines in Fig. 1) at the two drier sites.

**Table 5.** Simulated responses to increasing atmospheric CO_2_ concentrations (*C*_a_). First column in each block gives the actual values (for *C*_a_ = 317 ppm), while subsequent columns contain deviations (in %) from these values. Negative differences marked in red font. ‘Medium-term response’, constant vegetation properties (see Table 1) were optimized for *C*_a_ = 317 ppm; ‘Long-term adaptation’, all vegetation properties were optimized for the respective *C*_a_. *P*, precipitation; *Q*, drainage and runoff; *E*_T_, evapotranspiration (transpiration + soil evaporation)^1^; *E*_t_, transpiration^1^; *E*_s_, soil evaporation^1^; *G*_s_, big-leaf CO_2_ stomatal conductance^2^; WUE, water-use efficiency (total *A*_g_/total *E*_t_); iWUE, intrinsic WUE [average (*A*_g_/*G*_s_)]; *M*_A_, fractional cover of big leaf; *A*_g_, CO_2_ uptake rate^1^; *J*_max25_, leaf electron transport capacity^2^; *λ*_p_ and *λ*_s_, median of ∂*E*_t_/∂*A*_g_; RAI, root area index (fine root surface area per ground area); Θ_1_, soil saturation degree in top soil layer; Av(Θ), average saturation degree within the rooting zone of perennial vegetation. All magnitudes given as averages (*λ*_p_ and *λ*_s_: median values) over last 5 years of simulation. Note that at steady-state, total *P* = total *Q* + total *E*_T_. However, in the simulations for VIR, soil water storage (saturated + unsaturated) varies by up to 1000 mm on a decadal scale, and in fact decreased in the last 5 years of the simulation by roughly 500 mm, explaining the mean annual imbalance of 100 mm at this site (see Fig. 2 in the SI). ^1^Per m^2^ ground area; ^2^per m^2^ projected leaf area.

Variable *C*_a_	Units ppm	VIR			HS			TUM			CT		
		317	10.4	19.9	317	10.4	19.9	317	10.4	19.9	317	10.4	19.9
**Medium-term response**													
Total *P*	mm year^−1^	401	0.0	0.0	1630	0.0	0.0	1070	0.0	0.0	3280	0.0	0.0
Total *Q*	mm year^−1^	101	−1.7	−3.0	335	3.1	7.1	261	7.4	13.2	1320	3.6	6.9
Total *E*_T_	mm year^−1^	400	1.0	1.5	1320	−0.9	−1.8	883	−1.8	−3.5	1790	−2.5	−4.8
*E*_t,p_	mm year^−1^	102	1.2	1.5	611	−1.5	−2.9	227	−3.4	−6.1	456	−3.0	−5.8
*E*_t,s_	mm year^−1^	182	6.6	9.9	570	0.3	0.2	458	−2.2	−4.6	1040	−3.3	−6.2
*E*_s_	mm year^−1^	115	−8.1	−11.9	143	−2.9	−5.5	198	1.0	2.0	296	1.0	1.8
*G*_s,p_	mmol s^−1^	117	−2.0	−4.1	364	−2.5	−4.7	404	−5.3	−9.5	926	−4.1	−7.8
*G*_s,s_	mmol s^−1^	103	−2.4	−4.1	226	−3.7	−7.0	401	−5.4	−9.7	701	−4.3	−8.0
*M*_A,p_		0.22	0.0	0.0	0.30	0.0	0.0	0.28	0.0	0.0	0.24	0.0	0.0
*M*_A,s_		0.42	4.6	6.6	0.44	3.5	6.9	0.71	0.5	1.0	0.76	0.2	0.2
*A*_g,p_	mmol day^−1^	87.7	10.5	19.2	208	7.6	13.9	230	4.1	7.4	206	4.8	8.5
*A*_g,s_	mmol day^−1^	160	16.0	28.1	254	10.4	19.7	535	5.2	9.4	604	5.3	9.7
WUE_p_	mmol mol^−1^	5.63	9.2	17.5	2.24	9.2	17.3	6.65	7.7	14.3	2.97	8.1	15.2
WUE_s_	mmol mol^−1^	5.78	8.9	16.5	2.92	10.1	19.4	7.67	7.6	14.7	3.82	8.9	17.0
iWUE_p_	μmol mol^−1^	151	11.3	21.5	76.1	9.6	17.7	84.1	8.6	16.1	47.3	7.2	13.9
iWUE_s_	μmol mol^−1^	156	11.6	22.2	108	12.7	23.6	94.8	8.9	16.6	63.0	8.5	15.6
*J*_max25,p_	μmol s^−1^	257	4.9	8.6	351	2.6	4.7	809	1.0	2.1	501	1.2	2.1
*J*_max25,s_	μmol s^−1^	218	3.9	8.6	252	2.8	4.2	785	1.2	2.1	491	1.3	2.2
*λ*_p_	mol mol^−1^	287	−4.4	−7.4	2060	0.2	0.7	953	3.0	6.1	3670	5.2	9.2
*λ*_s_	mol mol^−1^	207	−3.3	−5.1	809	−1.3	−1.7	1190	3.3	6.5	2360	2.6	4.8
RAI_p_	m^2^ m^−2^	0.37	43.8	82.6	0.44	−4.0	−8.9	0.15	−7.8	−14.6	0.094	−4.8	−8.7
RAI_s_	m^2^ m^−2^	0.53	45.5	89.8	0.59	38.3	83.6	0.38	−2.4	−1.2	0.17	−7.6	−12.5
Θ_1_		0.11	−2.7	−4.9	0.20	−0.3	−0.3	0.41	1.3	2.7	0.54	1.1	2.0
Av(Θ)		0.20	−2.1	−3.3	0.24	0.7	1.7	0.50	0.9	1.8	0.61	0.7	1.4
**Long-term adaptation**													
Total *P*	mm year^−1^	401	0.0	0.0	1630	0.0	0.0	1070	0.0	0.0	3280	0.0	0.0
Total *Q*	mm year^−1^	101	−2.6	0.9	335	−1.1	−19.9	261	5.5	8.0	1320	1.7	2.3
Total *E*_T_	mm year^−1^	400	1.2	0.3	1320	0.2	5.9	883	−1.3	−2.1	1790	−1.1	−1.5
*E*_t,p_	mm year^−1^	102	10.1	13.1	611	5.9	26.2	227	−0.6	−0.8	456	−4.3	−2.4
*E*_t,s_	mm year^−1^	182	1.2	0.3	570	−5.4	−13.5	458	−2.3	−4.2	1040	−0.1	−1.8
*E*_s_	mm year^−1^	115	−6.6	−11.1	143	−2.1	−3.4	198	0.3	1.2	296	0.4	1.0
*G*_s,p_	mmol s^−1^	117	−0.0	−9.6	364	−1.8	−8.1	404	−5.5	−7.5	926	−4.6	−4.4
*G*_s,s_	mmol s^−1^	103	−3.6	−6.0	226	−6.1	−7.9	401	−4.4	−6.9	701	−0.7	−1.9
*M*_A,p_		0.22	9.3	25.6	0.30	7.3	32.8	0.28	3.4	4.7	0.24	−0.4	1.4
*M*_A,s_		0.42	2.7	5.2	0.44	0.7	−4.3	0.71	−0.5	−0.8	0.76	0.3	−0.2
*A*_g,p_	mmol day^−1^	87.7	18.3	39.6	208	14.7	51.0	230	7.4	12.2	206	4.2	9.9
*A*_g,s_	mmol day^−1^	160	12.0	22.1	254	6.7	5.6	535	4.3	7.6	604	5.7	9.4
WUE_p_	mmol mol^−1^	5.63	7.5	23.5	2.24	8.3	19.6	6.65	8.1	13.1	2.97	8.8	12.6
WUE_s_	mmol mol^−1^	5.78	10.7	21.6	2.92	12.9	22.1	7.67	6.7	12.3	3.82	5.8	11.4
iWUE_p_	μmol mol^−1^	151	10.4	23.9	76.1	10.9	19.3	84.1	8.9	15.7	47.3	8.7	13.9
iWUE_s_	μmol mol^−1^	156	11.9	22.7	108	13.2	23.1	94.8	8.2	15.1	63.0	7.2	13.6
*J*_max25,p_	μmol s^−1^	257	3.5	3.5	351	1.8	5.0	809	1.0	1.9	501	1.0	2.0
*J*_max25,s_	μmol s^−1^	218	3.8	8.1	252	2.2	3.5	785	1.4	2.1	491	1.5	2.3
*λ*_p_	mol mol^−1^	287	−6.5	−18.4	2060	0.9	−4.6	953	2.7	8.2	3670	2.6	11.1
*λ*_s_	mol mol^−1^	207	−7.3	−13.3	809	−6.7	−8.4	1190	6.0	13.5	2360	7.5	13.5
RAI_p_	m^2^ m^−2^	0.37	21.1	51.2	0.44	9.4	−15.7	0.15	−3.3	−8.1	0.094	−5.4	−5.9
RAI_s_	m^2^ m^−2^	0.53	8.6	7.2	0.59	11.8	−15.9	0.38	10.1	2.2	0.17	−3.2	−9.9
Θ_1_		0.11	0.7	3.8	0.20	1.1	4.9	0.41	1.1	1.9	0.54	0.5	1.2
Av(Θ)		0.20	−2.2	−0.9	0.24	−0.6	2.5	0.50	0.6	1.0	0.61	0.4	0.7

### Stomatal conductance, roots and evapotranspiration

**Figure 2.**
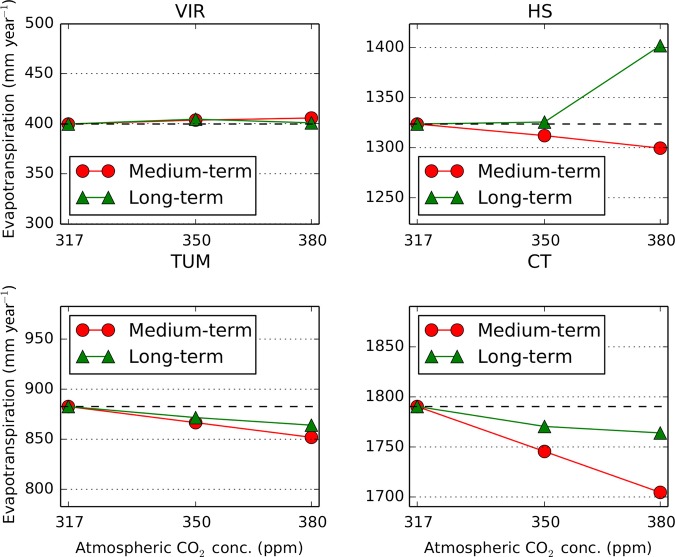
Simulated mean annual evapotranspiration rates for different atmospheric CO_2_ concentrations (*C*_a_). ‘Medium-term’ refers to simulations where constant vegetation properties (see Table 1) were optimized for *C*_a_ = 317 ppm, while dynamic vegetation properties were optimized for the respective *C*_a_. ‘Long-term’ refers to simulations where all vegetation properties were optimized for the respective *C*_a_. The horizontal black dashed lines are a visual guide to see the change relative to the *E*_T_ rates at 317 ppm *C*_a_.

In all simulations, stomatal conductance decreased in response to elevated CO_2_ concentrations (*C*_a_) (Table 5). Except for the driest site, the simulated medium-term response of evapotranspiration (*E*_T_, sum of transpiration by perennial and seasonal vegetation plus soil evaporation) was a decrease in the order of 10–80 mm year^−1^ for an increase in *C*_a_ of 63 ppm (Fig. 2). The simulated long-term response, in contrast, ranged from a slight decrease (10–25 mm year^−1^) to increases in *E*_T_ by up to 70 mm year^−1^ (Fig. 2) when increasing *C*_a_ from 317 to 380 ppm. The largest increase at the HS site was accompanied by an increase in perennial vegetation rooting depth (from 4.5 to 5 m) and *M*_A,p_ (from 0.3 to 0.39) in the model. For all other sites, simulated rooting depths of perennial vegetation were at 2 m and invariant (data not shown). For the driest site (VIR), simulated perennial FPC increased from 0.22 to 0.28, with a corresponding increase in transpiration by perennial plants (*E*_t,p_ in the second part of Table 5), but this was accompanied by a decrease in transpiration by seasonal vegetation and soil evaporation (*E*_t,s_ and *E*_s_, respectively, in the second part of Table 5), resulting in a very small sensitivity of total *E*_T_ to *C*_a_ at this site. In general, the predictions considering long-term adaptation led to higher *E*_T_ rates than those considering medium-term adaptation only (Fig. 2). Simulated root area indices (RAI) had a tendency to increase with *C*_a_ in the water-limited catchments and to decrease in the energy-limited catchments. The increases in RAI with *C*_a_ at the water-limited sites were much more pronounced in the medium-term adaptation scenario (up to 100 % increase) than for long-term adaptation (up to 25 % increase, Table 5).

#### Medium-term simulations

**Figure 3.**
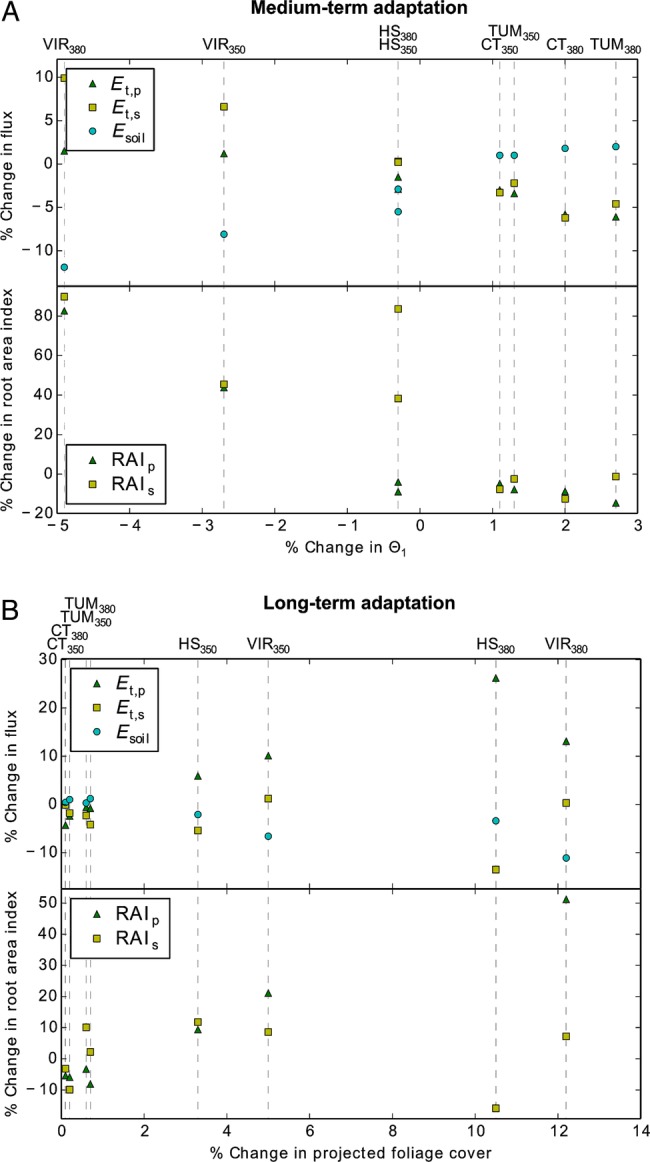
Relative changes in evaporative fluxes and RAI vs. relative changes in (A) surface soil moisture for medium-term and (B) FPC in long-term adaptation. *E*_t_, transpiration; *E*_s_, soil evaporation; Θ_1_, relative saturation in the top 0.5 m of soil; RAI, root area index. Subscripts p and s refer to perennial and seasonal vegetation, respectively. Dashed lines link points belonging to a given site (codes following Table 2) and atmospheric CO_2_ concentration (subscripts to side codes).

Transpiration responses were partly offset by opposite responses in soil evaporation, which was strongly correlated with surface soil moisture (Θ_1_, top 50 cm, Fig. 3A). Figure 3A also illustrates that in the medium-term simulations, surface soil moisture decreased at the driest site (VIR), changed very little at the intermediate site (HS) and increased at the energy-limited sites (TUM and CT) in response to increasing *C*_a_. Simulated trends in root area indices (RAI_p_ and RAI_s_ for perennial and seasonal vegetation, respectively) were relatively similar to those in transpiration rates, except at HS, where a strong increase in RAI_s_ coincided with little change in *E*_t,s_ (Fig. 3A).

#### Long-term simulations

Simulated soil moisture increased slightly at all sites (all changes <5 %, Table 5). Here, soil evaporation decreased with increasing FPC, in favour of increasing transpiration by perennial plants (*E*_t,p_), while transpiration by seasonal plants (*E*_t,s_) did not show very clear trends (Fig. 3B). Root area indices (RAI_p_ and RAI_s_) again show similar trends to transpiration rates, with an exception at HS, where the largest increase in *E*_t,p_ was accompanied by a decrease in RAI_p_.

### Photosynthesis and water-use efficiency

Table 5 and Fig. 3A and B show the following additional trends:

1. Photosynthetic capacities (*J*_max25_) and CO_2_ assimilation rates (*A*_g_) increased with *C*_a_ in all simulations, with a stronger increase in *A*_g_ for seasonal vegetation in the medium term, but a stronger increase for perennial vegetation in the long term. These trends were consistent across all four sites.
2. Water-use efficiency [both WUE and intrinsic WUE (iWUE)] were generally lower for perennial compared with seasonal vegetation at each site, and increased linearly with increasing atmospheric CO_2_ at all sites with up to 24 % increase at 380 ppm compared with 317 ppm, both under medium and long-term adaptation.
3. The median values of the slope between CO_2_ uptake and transpiration (*λ* = ∂*E*_t_/∂*A*_g_) give an indication whether water is used more or less conservatively. The lower the values of *λ*, the more water use is limited to times favourable for higher WUE (e.g. high relative humidity of the air and light availability) and hence the more conservative the water use. Simulated *λ*_p_ and *λ*_s_ (for perennial and seasonal vegetation, respectively) generally decreased with increasing *C*_a_ at the water-limited sites (VIR and HS), while they increase with *C*_a_ at the energy-limited sites (TUM and CT). This effect is slightly more pronounced under long-term adaptation but equally clear under medium-term adaptation.

Direct comparison of relative changes in response to eCO_2_ under medium and long-term adaptation scenarios reveals clear differences only for drainage (*Q*), total evapotranspiration (*E*_T_), transpiration by perennial and seasonal vegetation (*E*_t,p_ and *E*_t,s_, respectively), FPC, CO_2_ assimilation rate (*A*_g_), the water-use strategy indicator *λ* and RAI, as summarized in Table 6. As mentioned before, *E*_T_ was generally higher under long-term adaptation and hence drainage was lower, compared with medium-term adaptation. This was largely caused by a stronger increase in FPC of perennial plants under long-term adaptation. CO_2_ assimilation rates (*A*_g_) increased stronger than FPC, and generally stronger under long-term adaptation. Root area index was generally lower under long-term adaptation at the two water-limited sites (VIR and HS).

**Table 6.** Relative CO_2_ sensitivities in medium and long-term response scenarios derived from Table 5. Values indicate relative change per relative change in *C*_a_ as *C*_a_ was increased from 317 to 380 ppm. A value of, for example, 0.7 indicates that the relative response of this variable was 70 % of the relative change in *C*_a_, i.e. 14 % increase for a 20 % increase in *C*_a_. Negative values marked in red font. ‘med.’, constant vegetation properties (see Table 1) were optimized for *C*_a_ = 317 ppm; ‘long’, all vegetation properties were optimized for *C*_a_ = 380 ppm.

	VIR		HS		TUM		CT	
	Medium	Long	Medium	Long	Medium	Long	Medium	Long
Total *Q*	−0.2	0.0	0.4	−1.0	0.7	0.4	0.3	0.1
Total *E*_T_	0.1	0.0	−0.1	0.3	−0.2	−0.1	−0.2	−0.1
*E*_t,p_	0.1	0.7	−0.1	1.3	−0.3	−0.0	−0.3	−0.1
*E*_t,s_	0.5	0.0	0.0	−0.7	−0.2	−0.2	−0.3	−0.1
*E*_s_	−0.6	−0.6	−0.3	−0.2	0.1	0.1	0.1	0.1
*G*_s,p_	−0.2	−0.5	−0.2	−0.4	−0.5	−0.4	−0.4	−0.2
*G*_s,s_	−0.2	−0.3	−0.4	−0.4	−0.5	−0.3	−0.4	−0.1
FPC_p_	0.0	1.3	0.0	1.6	0.0	0.2	0.0	0.1
FPC_s_	0.3	0.3	0.3	−0.2	0.1	−0.0	0.0	−0.0
*A*_g,p_	1.0	2.0	0.7	2.6	0.4	0.6	0.4	0.5
*A*_g,s_	1.4	1.1	1.0	0.3	0.5	0.4	0.5	0.5
WUE_p_	0.9	1.2	0.9	1.0	0.7	0.7	0.8	0.6
WUE_s_	0.8	1.1	1.0	1.1	0.7	0.6	0.9	0.6
iWUE_p_	1.1	1.2	0.9	1.0	0.8	0.8	0.7	0.7
iWUE_s_	1.1	1.1	1.2	1.2	0.8	0.8	0.8	0.7
*J*_max25,p_	0.4	0.2	0.2	0.3	0.1	0.1	0.1	0.1
*J*_max25,s_	0.4	0.4	0.2	0.2	0.1	0.1	0.1	0.1
*λ*_p_	−0.4	−0.9	0.0	−0.2	0.3	0.4	0.5	0.6
*λ*_s_	−0.3	−0.7	−0.1	−0.4	0.3	0.7	0.2	0.7
RAI_p_	4.2	2.6	−0.4	−0.8	−0.7	−0.4	−0.4	−0.3
RAI_s_	4.5	0.4	4.2	−0.8	−0.1	0.1	−0.6	−0.5
Θ_1_	−0.2	0.2	−0.0	0.2	0.1	0.1	0.1	0.1
Av(Θ)	−0.2	−0.0	0.1	0.1	0.1	0.1	0.1	0.0

Table 6 also reveals that, at the drier sites, simulated vegetation used more water under eCO_2_, which was partly compensated for by decreases in soil evaporation, while at the wetter sites, the changes were reversed. CO_2_ assimilation (*A*_g_) at the drier sites also benefited more from eCO_2_ than at the wetter sites, translating into an almost proportional scaling of WUE with atmospheric CO_2_ in the simulations (relative sensitivities ranging between 0.6 for the wettest site and 1.2 for the driest site).

## Discussion

The model presented here and its components have been tested at the HS site in previous publications (Schymanski *et al*. 2007, 2008*a*, *b*, 2009*b*) and further detailed tests are required before projections into the future are attempted. The simulations presented in this paper are intended as answers to the question what might be the response of vegetation to eCO_2_ at different temporal and spatial scales *IF* vegetation were to adapt in a way to maximize its NCP in the long term. In other words, the model is used as a tool to understand the implications of an optimality hypothesis on possible responses of vegetation to eCO_2_ in different climates. Therefore, no attempts were made to improve any of the model results by parameter tuning or changes to the model structure. In that respect, one advantage of optimality-based models is that they simulate the adaptation of plants to their environment based on a principle that is not expected to change as the environment changes and hence their performance for predictions into the future is not expected to be fundamentally worse than that for predicting the past.

### Ecological relevance of community-scale optimality

The origins of the community-scale optimality hypothesis adopted in the VOM can be traced back to Lotka (1922), who proposed that natural selection would yield communities that maximize their throughput of energy, Odum and Pinkerton (1955) who postulated that climax communities balance their primary productivity and maintenance cost while maximizing their biomass and Odum (1969), who extended the hypothesized goal of succession to include preservation of nutrients and protection from external perturbations by complex interactions including mutualism, commensalism and others. Maximization of the NCP can be seen as an approach to quantify the maximum amount of energy (in the form of assimilated carbon) that can be available within the ecosystem to support any such processes. It can been argued that natural selection does not act at the ecosystem level but instead acts on individuals and it has been shown on theoretical grounds that optimal resource use by competing plants is not equivalent to optimal resource use by a community of plants (Cowan 1982). However, consideration of more complex interactions in ecosystems resulted in the emergence of system-wide extrema in productivity or resource use (Loreau 1998) while the debate about the effects of natural selection at higher organizational levels is ongoing (Fussmann *et al*. 2007; Frank 2013; Pruitt and Goodnight 2014). A discussion of the consistency of optimality hypotheses with ecological theories is beyond the scope of this paper, but an overview of different optimality approaches relevant to ecohydrology can be found in Schymanski *et al*. (2009*a*).

### Some consequences of simplifying assumptions

The results presented here revealed that the model does not reproduce the correct partitioning between perennial and seasonal vegetation at the two energy-limited sites (CT, TUM) examined. This is likely due to the fact that the only advantage of perennial vegetation in the present model is the ability to develop root systems deeper than 1 m. When rainfall is abundant throughout the year, and deep roots are not useful for increasing the long-term NCP, seasonal vegetation would have an additional advantage in the model of being able to reduce their FPC and associated maintenance costs on the rare and short occasions of insufficient water availability and be favoured over perennial vegetation. More realistic partitioning could likely be achieved if the advantage of being tall for light capture was considered in the model. Given that total FPC fell only very rarely below 0.99 at the wet sites (TUM and CT, Fig. 1) we expect that any such modifications would just shift the dominance from seasonal to perennial vegetation in the simulations, but not affect the overall fluxes very much. As discussed below, the big-leaf simplification complicates comparisons between simulated and observed leaf-scale properties. These may be further complicated by the neglect of carboxylation-limited photosynthesis in the VOM. Both simplifications were adopted to reduce computational burden in a model where optimal adaptation is computed using a large number of model runs. Given the prior performance of the model and its components (Schymanski *et al*. 2007, 2008*a*, *b*, 2009*b*; Lei *et al*. 2008), we assume that the structure of the costs and benefits of the optimized vegetation properties is captured adequately despite the simplifications.

### Effect of spatial scale on eCO_2_ responses

The effect of eCO_2_ on evapotranspiration (*E*_T_) has been known to decrease with increasing scale from leaf to canopy to catchment (Leuzinger *et al*. 2011). Our modelling results identify several mechanisms that may be responsible for this scale effect by buffering the leaf-scale reduction in transpiration at larger scales. First and most importantly, the reduction in leaf-scale transpiration with increasing atmospheric CO_2_ causes more water to remain in the soil, which can either drain away and contribute to stream flow (as assumed in most papers mentioned in the introduction) or be utilized by additional leaves or plants, especially in water-limited environments. The latter is supported by the general increase in simulated average FPC (Table 5), consistent with observational data compiled by Norby and Zak (2011), expressing the strongest increase in leaf area index for sites with initially low leaf area index and in line with conclusions drawn from remote sensing data by Donohue *et al*. (2013). In addition to increased drainage and/or FPC, the increase in soil moisture resulting from a decrease in transpiration may result in increased soil evaporation (*E*_s_). This is indicated in the simulations for the energy-limited sites TUM and CT in Table 5, where both simulated soil moisture and soil evaporation rates increased with increasing *C*_a_. However, in the simulations for the water-limited sites (VIR and HS), increased transpiration and associated decreases in soil moisture and/or increases in ground shading due to increased FPC led to an overall reduction in soil evaporation in response to eCO_2_. In fact, the control on soil evaporation at the two water-limited sites shifted from soil moisture feedback in the medium-term simulations (Fig. 3A) to foliage cover feedback in the long-term simulations (Fig. 3B), whereas soil evaporation remained soil moisture controlled at the energy-limited sites (Table 5), where total FPC was close to 1 in all simulations (Fig. 1).

### Long-term vs. medium-term responses

In addition to the dampening of leaf-scale reductions in transpiration at larger spatial scales, our modelling results also suggest a clear time-scale dependency at some of the sites and for some variables. Except for the driest site (VIR), the simulations representing medium-term adaptation, i.e. where slowly varying vegetation properties (years-decades) were kept constant, showed a stronger reduction in evapotranspiration (*E*_T_) at eCO_2_ than simulations representing long-term adaptation, i.e. where all vegetation properties were optimized to the respective *C*_a_. In fact, at one of the sites (HS), long-term adaptation was predicted to lead to a dramatic reversal from an initial decrease in *E*_T_ by 30 mm year^−1^ in the medium-term to an increase by 100 mm year^−1^ in the long term, mainly caused by an increase in perennial vegetation cover and maximum rooting depth (Fig. 2). Except for the wettest site, there was a general shift in water use from seasonal to perennial vegetation between the medium-term and long-term adaptation simulations. Interestingly, the simulations showed a stronger increase in CO_2_ assimilation in response to eCO_2_ for seasonal plants compared with perennial plants in the medium-term, but a dramatic reversal, i.e. much stronger increases for perennial plants when long-term adaptation was considered. This was mainly due to increases in FPC for perennial plants in the long-term adaptation simulations, which were not permitted by design in the medium-term adaptation simulations. This implicates eCO_2_ as a direct contributor to ‘woody thickening’. In the present model, establishment of saplings and the effect of fires were not considered, so the reasons for the woody encroachment simulated here must be different from those suggested by Bond and Midgley (2000). Since all photosynthesis was modelled as C3 photosynthesis, the reasons for the woody thickening emerging from the model runs are also not related to physiological differences between woody C3 and herbaceous C4 plants, as suggested by Higgins and Scheiter (2012). In the VOM, perennial vegetation has the advantage of potential access to deeper soil water and can freely expand its FPC, whereas seasonal vegetation has a fixed rooting depth of 1 m and cannot exceed an FPC fraction of 1 minus the fractional cover of perennial vegetation. By its effect on WUE, eCO_2_ acts to shift water-limited environments more towards energy-limited conditions, where the usually taller perennial plants have a selective advantage.

### Effect of eCO_2_ on stomatal control

**Figure 4.**
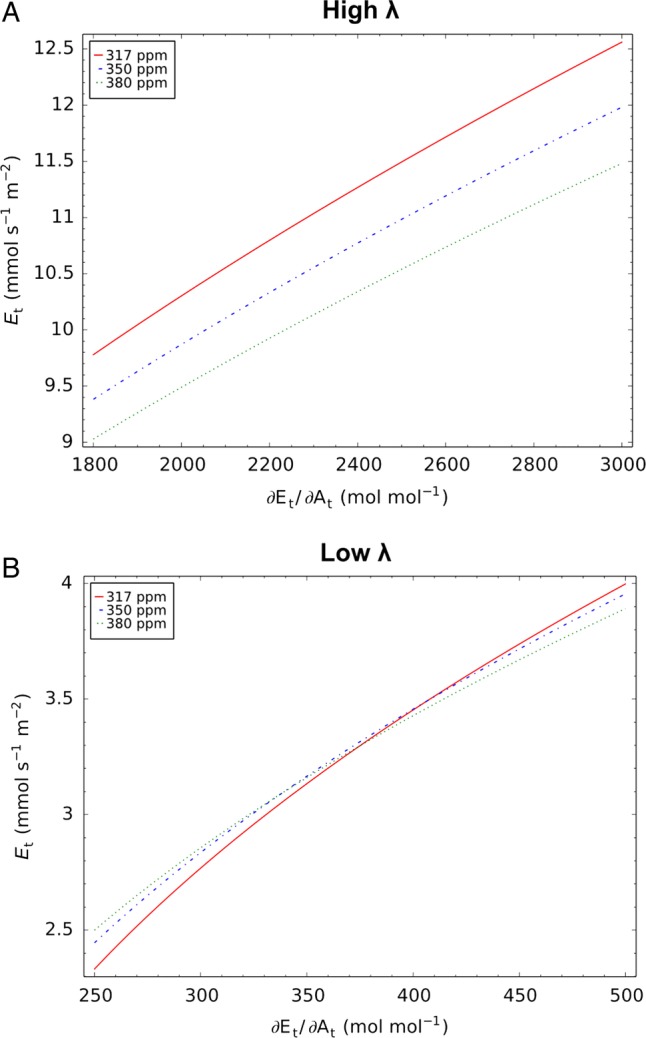
Sensitivity of transpiration rate (*E*_t_, per unit leaf area) to *λ* = ∂*E*_t_/∂*A*_g_ for different atmospheric CO_2_ concentrations (see keys) at high *λ* (A) and low *λ* (B). Simulation conditions: 1000 μmol m^−2^ s^−1^ PPFD, 0.02 mol H_2_O mol^−1^ air vapour deficit (equivalent to 2 kPa VPD), 40 ppm Γ_*_. Ranges of *λ* and *J*_max_ in (A) and (B) represent simulated values at the wettest site (CT, *J*_max_ = 485 μmol m^−2^ s^−1^) and the driest site (VIR, *J*_max_ = 250 μmol m^−2^ s^−1^), respectively (see Table 5).

An apparently paradox result is the negative correlation between trends in *λ* = ∂*E*/∂*A* and RAI. At the water-limited sites, *λ* decreases with increasing *C*_a_ (indicating more conservative water use), but RAI increases, while at the energy-limited sites, *λ* increases under eCO_2_ (indicating less conservative water use) while RAI decreases. This can be better understood when looking at the combined effects of changes in *C*_a_ and *λ* on transpiration rates. If everything else stays constant, increasing values of *λ* increase transpiration rates. However, at high constant values of *λ*, eCO_2_ would commonly reduce transpiration rates (Fig. 4A) while at low constant values of *λ*, eCO_2_ could also lead to an increase in transpiration rates (Fig. 4B). This is due to the non-linear effect of *C*_a_ on the shape of the *A*_g_(*g*_s_) curve and hence on the slope *λ* = ∂*E*/∂*A*. In the medium-term adaptation simulations, *λ* only responds to changes in soil moisture, i.e. reduction in transpiration under eCO_2_ leads to increased soil moisture and hence increased *λ* and transpiration rates, representing a negative feedback loop. Conversely, increase in transpiration under eCO_2_ at low values of *λ* would decrease soil moisture and hence decrease *λ*, resulting again in a negative feedback loop. In the long-term adaptation simulations, the response of *λ* to soil moisture is optimized for maximum NCP, along with all other vegetation properties. The results suggest that *λ* should not be expected to scale with *C*_a_ in any straightforward way, as assumed by, for example, Katul *et al*. (2010), but is only part of the whole plant adaptation to its environment (Buckley and Schymanski 2014).

### Effect of eCO_2_ on photosynthesis and WUE

Another apparently paradoxical result is that, contrary to previously employed approaches (e.g. Prentice *et al*. 2011), where the assumption of constant *c*_i_/*c*_a_ ratios resulted in decreased biochemical capacity (expressed as *V*_cmax_) in response to eCO_2_, the simulations here all resulted in increasing biochemical capacity (*J*_max25_) with increasing *c*_a_. This is in contrast to general findings in FACE studies, where leaf-scale biochemical parameters, *J*_max_ and *V*_cmax_, have been found to decrease under eCO_2_ (Ainsworth and Long 2005). This observed down-regulation of photosynthetic capacity is generally attributed to nutrient limitation or insufficient carbon sink capacity (Ainsworth and Long 2005; Leakey *et al*. 2009), neither of which are considered in the VOM. In contrast, the VOM results suggest that higher net carbon uptake rates could be achieved with higher *J*_max25_ under eCO_2_. In this context, it is important to consider that the VOM simulates the properties of a ‘big leaf’, representing aggregated canopy properties rather than leaf-scale properties, whereas decreases in *J*_max_ under eCO_2_ have been reported at the leaf scale (see above), with a simultaneous increase in leaf area index, even for relatively closed canopies (Norby and Zak 2011). These simultaneous responses may well have led to an overall increase in canopy-scale photosynthetic capacity (as predicted in the present study) despite decreases at the leaf scale. Note that the big-leaf representation of the canopy and the neglect of nutrient limitation are also features of many global scale models (e.g. Prentice *et al*. 2011), so their apparent consistency with leaf-scale observations of decreasing *V*_cmax_ and/or *J*_max_ under eCO_2_ may not be a good indication for the correct representation of acclimation to eCO_2_.

The most coherent response to eCO_2_ across all simulations is an increase in WUE and iWUE (assimilation rate divided by stomatal conductance), with relative responses varying between 0.6 and 1.2 (Table 6), i.e. roughly doubling WUE for a doubling in atmospheric CO_2_ concentration. This coincides with the range observed using FACE (Table 7). In a study focussing on two FACE sites (Duke and Oak Ridge), De Kauwe *et al*. (2013) found that 11 state of the art process-based ecosystem models produced relative responses of WUE to eCO_2_ between 0.24 and 0.88 while the observed relative responses were 0.65 and 0.93 at the two sites. It is remarkable that the unmodified optimality model employed here produced such a robust eCO_2_ sensitivity across four very contrasting catchments, that was in close agreement with general trends in FACE results, while more empirically based models with direct parametrizations of stomatal sensitivity to eCO_2_, partly based on other FACE experiments, produced much more scatter with a tendency to under-estimate the response of WUE to eCO_2_.

**Table 7.** Documented vegetation responses to eCO_2_ vs. model predictions. Relative responses were deduced from reported relative change in vegetation property divided by relative change in *C*_a_ (e.g. for FACE experiments running at 580 ppm, relative change in *C*_a_ would be 580/380 − 1 = 0.5. FACE, free-air CO_2_ enrichment; WTC, whole tree chamber. Sources: ^1^Ainsworth and Long (2005), ^2^Norby and Zak (2011), ^3^Franks *et al*. (2013), ^4^Ainsworth and Rogers (2007), ^5^Iversen (2010), ^6^Ferguson and Nowak (2011), ^7^Barton *et al*. (2012), ^8^De Kauwe *et al*. (2013), ^9^Tausz-Posch *et al*. (2013), ^10^Battipaglia *et al*. (2013).

Property	Observed relative response	Source	Predicted relative response	
			Medium	Long
Stomatal conductance	−0.2 to −0.7	FACE^1,2,3,4^	−0.2 to −0.5	−0.1 to −0.5
LAI	0 to +1	FACE^2^	0 to +0.3	0 to +1.6
Tree rooting depth	0/+	FACE^2,5,6^	N/A	0 to 0.6
Fine roots	+/−	FACE^2,6^	−0.7 to +4.5	−0.8 to +2.6
Soil moisture	+	FACE^2^	−0.2 to +0.1	0 to +0.1
WUE	+0.7 to +1.4	FACE and WTC^7,8,9^	+0.7 to +1.0	+0.6 to +1.2
iWUE	+1 to +1.8	FACE^1,9,10^	+0.7 to +1.2	+0.6 to +1.2

### Synthesis and comparison with FACE results

**Figure 5.**
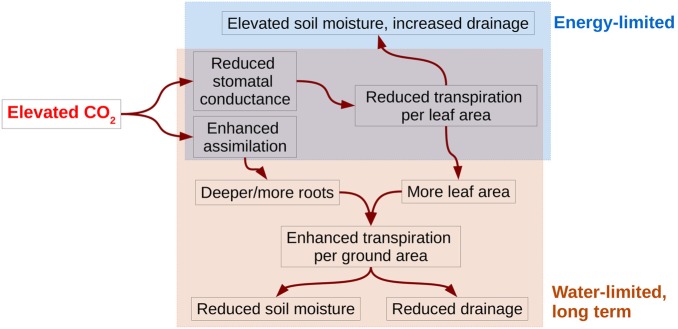
Summary of effects of eCO_2_ on vegetation and water resources for constant climate. Effects specific to either water-limited or energy-limited catchments are in the respective coloured boxes. Note that decrease in transpiration per unit leaf area has an initial effect on increasing soil moisture in all catchments, whereas initially increased soil moisture and enhanced assimilation results in increasing leaf area and increased transpiration per ground area at the water-limited sites, reversing the initial effect on soil moisture.

**Figure 6.**
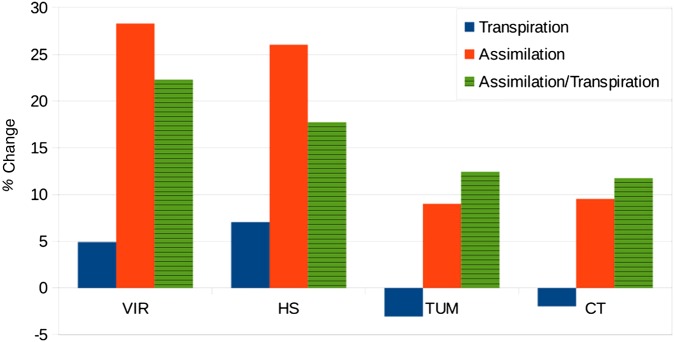
Relative response of transpiration, CO_2_ assimilation and their ratio to a 20 % increase in atmospheric CO_2_ concentrations at constant climate, assuming long-term adaptation.

In summary, the results presented in this study suggest that the primary effects of eCO_2_ are a reduction of stomatal conductance and an enhancement of CO_2_ assimilation. The former leads to reduced transpiration per leaf area and an initially elevated soil moisture (Fig. 5), leading to increased drainage in energy-limited catchments. However, in water-limited catchments, elevated soil moisture is likely to result in increasing leaf area, while enhanced assimilation allows for the production of more and deeper roots, all of which would act to allow the vegetation to increase the light absorption by the canopy. The net effect is to either maintain, or even enhance, transpiration per unit ground area and to reduce soil moisture and drainage. Note that the relative increase in assimilation per unit ground area is very similar to relative increase in atmospheric CO_2_ concentrations at the dry sites (VIR, HS) but at the wet sites, the response in assimilation to a 20 % increase in atmospheric CO_2_ is more than halved, at around 10 % (Fig. 6).

The recent review of the FACE literature by Norby and Zak (2011) reveals that many of the long-term effects of eCO_2_ predicted in the present study have already been observed experimentally, most notably increases in leaf area index, tree rooting depths and soil moisture. The observed responses are summarized in Table 7, which also indicates that the simulations presented here, both the medium and long-term adaptation scenarios, correspond very closely to general trends observed in FACE experiments. This is particularly remarkable given that most of the FACE data stems from experiments in temperate climates and managed ecosystems, whereas our model simulations refer to natural vegetation in semi-arid to tropical ecosystems. Both model simulations and FACE results illustrate a remarkable convergence in some vegetation responses to eCO_2_ across biomes and time scales, such as decreasing stomatal conductance, increasing WUE and in dry regions at least, an increase in the leaf area index.

## Conclusions

The present analysis of the effects of eCO_2_ on the economics of vegetation water use and carbon gain suggests that the assumption of optimal vegetation leads to results that are similar to observed patterns. From those results we also conclude that eCO_2_ may be responsible for a large part of the globally observed shift towards more perennial vegetation (‘woody thickening’). The study provides theoretical support for an eCO_2_-vegetation feedback that has the capacity to dampen reductions in vegetation water use as a result of stomatal down-regulation by allowing more leaf area and/or plants to thrive in water-limited environments. Considering different time scales of adaptation for different vegetation properties, the study separates responses to eCO_2_ likely occurring at different temporal scales and suggests that reductions in water use due to stomatal down-regulation should be expected in the shorter term, while unchanged or even increased water use due to an increase in leaf area, plant abundance and potentially rooting depths may result in the longer term in water-limited systems. This suggests that the still common assumption that eCO_2_ will generally reduce vegetation water use due to reductions in leaf-level stomatal conductance is not justified in water-limited catchments.

## Sources of Funding

This study was supported by Swiss National Science Foundation (S.J.S.: 200021-113442) and Australian Research Council (S.J.S.: DP120101676, M.L.R.: CE11E0098).

## Contributions by the Authors

S.J.S. conceived and carried out the research, analysed the data and wrote the text in frequent collaboration with M.L.R. M.S. contributed thoughts and ideas, and helped develop and focus the text at various stages.

## Conflict of Interest Statement

None declared.

## Supporting Information

The following additional information is available in the online version of this article –

**File S1.** A more detailed description of the VOM and modification applied in this study.
